# Enhancing Automated Breast Cancer Detection: A CNN-Driven Method for Multi-Modal Imaging Techniques

**DOI:** 10.3390/jpm15100467

**Published:** 2025-10-01

**Authors:** Khadija Aguerchi, Younes Jabrane, Maryam Habba, Mustapha Ameur, Amir Hajjam El Hassani

**Affiliations:** 1 Cadi Ayyad University, UCA, ENSA, Modeling and Complex Systems (LMSC), P.O. Box 575, Av. Abdelkrim Khattabi, Marrakech 40000, Morocco; khadija.aguerchi@edu.uca.ma (K.A.); m.habba@uca.ac.ma (M.H.); m.ameur@uca.ac.ma (M.A.); 2 Marie and Louis Pasteur University, Belfort Montbéliard University of Technology, SINERGIES, F-90000 Belfort, France; amir.hajjam-el-hassani@utbm.fr

**Keywords:** computer-aided diagnosis, deep learning, CNN, imaging modalities, mammography, magnetic resonance imaging, ultrasound, histopathology

## Abstract

**Background/Objectives:** Breast cancer continues to be one of the primary causes of death among women worldwide, emphasizing the necessity for accurate and efficient diagnostic approaches. This work focuses on developing an automated diagnostic framework based on convolutional neural networks (CNNs) capable of handling multiple imaging modalities. **Methods:** The proposed CNN model are trained and evaluated on several benchmark datasets, including mammography (DDSM, MIAS, INbreast), ultrasound, magnetic resonance imaging (MRI), and histopathology (BreaKHis). Standardized preprocessing procedures were applied across all datasets, and the outcomes were compared with leading state-of-the-art techniques. **Results:** The model attained strong classification performance with accuracy scores of 99.2% (DDSM), 98.97% (MIAS), 99.43% (INbreast), 98.00% (Ultrasound), 98.43% (MRI), and 86.42% (BreaKHis). These findings indicate superior results compared to many existing approaches, confirming the robustness of the method. **Conclusions:** This study introduces a reliable and scalable diagnostic system that can support radiologists in early breast cancer detection. Its high accuracy, efficiency, and adaptability across different imaging modalities make it a promising tool for integration into clinical practice.

## 1. Introduction

Breast cancer is the second leading cause of cancer-related mortality worldwide, following lung cancer [1]. Without early detection, it represents a major source of female mortality [2]. Conversely, timely diagnosis significantly improves survival rates, as breast cancer is among the most treatable cancers when detected early [3], reducing both mortality and patient distress [4]. Various imaging modalities, including digital mammography, ultrasound, MRI, and histopathology, are widely used for the early detection and diagnosis of breast cancer [5].

Digital mammography remains the primary screening tool, producing high-resolution images to identify calcifications and masses, yet its performance declines in dense breast tissue. Ultrasound is widely used to distinguish solid from cystic lesions and to guide biopsies, while MRI is particularly valuable for high-risk patients and ambiguous cases. Despite their utility, these modalities suffer from variability in interpretation and intrinsic limits of sensitivity and specificity, often leading to false positives or negatives [6]. These constraints highlight the need for advanced diagnostic approaches to improve accuracy and reliability.

Artificial intelligence (AI) has emerged as a transformative solution in medical imaging, capable of analyzing large datasets such as mammograms [7] and MRIs with performance comparable to or exceeding human experts [8,9]. By detecting subtle patterns beyond human perception [9], AI has proven effective in diverse applications, including skin leishmaniasis [10] and breast cancer detection [11].

However, most computer-aided diagnosis (CAD) systems remain modality-specific, limiting adaptability and requiring multiple frameworks to handle mammography, ultrasound, MRI, or histopathology. To overcome these challenges, we propose a unified convolutional neural network (CNN) framework capable of analyzing multiple modalities within a single model. This modality-agnostic approach leverages shared imaging patterns, streamlining deployment, reducing costs, and improving flexibility, while maintaining robust diagnostic performance across imaging types.

Deep learning, particularly deep convolutional neural networks (DCNNs), has demonstrated remarkable success in medical image analysis by automatically extracting complex features [12], surpassing traditional machine learning methods that rely on manual feature engineering [13].

The objective of this study is to develop a unified, multimodal CNN framework that minimizes overfitting through optimized architecture design, enhances diagnostic accuracy, and eliminates manual feature extraction. By integrating multiple imaging modalities with deep learning, the proposed system aims to advance breast cancer diagnosis, enable earlier detection, and ultimately improve patient outcomes.

## 2. Related Works

Machine learning (ML) and deep learning (DL) have been extensively applied to breast cancer diagnosis, primarily for binary or multi-class classification, evaluated using metrics such as accuracy, precision, recall, and F1 score. CNN-based CAD systems offer faster, more reliable detection across modalities including ultrasound, MRI, X-ray, and mammography [14], with AI in digital mammography and tomosynthesis matching or exceeding conventional CADe/CADx performance [15]. DL also enables analysis of genetic and histopathological data for early detection, supporting timely diagnosis and improved outcomes [16]. AI-assisted mammography improves detection and reduces radiologist workload despite challenges such as false positives and variable subgroup performance [17], and AI-powered CAD has notably enhanced mammography accuracy, showing strong potential for future breast cancer screening [18].

Mammogram Images: DCNNs with preprocessing and augmentation effectively classify mammograms into benign, malignant, and normal while handling class imbalance [19]. Castro-Tapia et al. [20] used segments from the INbreast and MIAS datasets to analyze and compare breast lesion classification architectures such as AlexNet, GoogleNet, VGG19, and ResNet50. In line with previous studies, they evaluated 14 malignant and benign microcalcification and mass classifiers from prior research. CNNs yielded exceptional results. GoogleNet emerged as the most accurate model in their CAD system for breast cancer diagnosis, achieving an F1 score of 91.92%, an AUC of 99.29%, a precision rate of 92.15%, an accuracy rate of 91.92%, a specificity rate of 97.66%, and a sensitivity rate of 91.70% on a balanced dataset. Chougrad et al. [21] developed a CNN-based breast cancer screening method to enhance mammographic image classification in the DDSM dataset.

These studies include: Rahman et al. [22], who employed pre-trained convolutional neural network (CNN) architectures, specifically ResNet50 and InceptionV3, to categorize mammographic lesions into benign and malignant classifications. Due to the limited availability of data, methods such as data augmentation, preprocessing, and transfer learning were implemented, with some approaches also incorporating encoder mechanisms. The ResNet50 model achieved an accuracy of 85.7%, while InceptionV3 recorded an accuracy of 79.6%.

Sun et al. [23] integrated features derived from multiple perspectives (MLO and CC) within the convolutional neural network framework. This model introduced a penalty term and utilized features across various scales, resulting in an accuracy of 82.02%. In [24], Jafari et al. extracted features from several pre-trained CNN models to identify breast cancer. The most relevant features were selected using mutual information and classified using neural networks (NN), k-nearest neighbors (kNN), random forests (RF), and support vector machines. This novel approach achieved an accuracy of 92% on the RSNA dataset, 94.5% on MIAS, and 96% on DDSM.

Muduli et al. [25] developed a CNN with five learnable layers (four convolutional and one fully connected) for breast cancer classification. The model automates feature extraction with fewer parameters and was rigorously tested on multiple mammography and ultrasound datasets (MIAS, DDSM, INbreast, BUS-1, BUS-2). It outperformed several methods, achieving 96.55%, 90.68%, and 91.28% accuracy on the MIAS, DDSM, and INbreast datasets, and 100% and 89.73% accuracy on the BUS-1 and BUS-2 datasets.

A hybrid mammography computer-aided diagnosis (CADx) system was developed by Rouhi and Jafari in [26], integrating region-based, contour-based, and clustering segmentation methodologies. The system employs spatial frequency components (SFC), enhanced region growing (RG), or convolutional neural networks (CNN) for preliminary segmentation and utilizes genetic algorithm-artificial neural networks (GA-ANN) or genetic algorithm-multiple artificial neural networks (GA-MA-ANN) for the optimization of level set parameters. Neoplasms were categorized using classifiers, including artificial neural networks (ANN), random forests, and support vector machines (SVM), achieving high levels of sensitivity, specificity, accuracy, and area under the curve (AUC) across various datasets (MIAS, DDSM, INbreast). The multilayer perceptron (MLP) classifier achieved accuracies of 90.94%, 88.61%, and 89.23% for the aforementioned datasets, respectively.

A novel feature extraction technique based on the Dual Contourlet Transform (Dual-CT) was introduced by Dong et al. in [27] for breast cancer diagnosis. In conjunction with an enhanced k-nearest neighbor (kNN) classifier, the methodology involved extracting regions of interest (ROI) from the MIAS database, followed by decomposition using Dual-CT, contourlet, and wavelet transforms, and extracting texture features. This approach achieved classification accuracies of 94.14% and 95.76%, surpassing conventional techniques. The enhanced kNN classifier demonstrated accuracies of 95.76%, 86.54%, and 89.30% for the MIAS, DDSM, and INbreast datasets, respectively.

Aguerchi et al. [11] conducted a study that employed transfer learning methodologies through the application of pre-trained convolutional neural network (CNN) architectures, specifically VGG16, ResNet50, and InceptionV3, to classify mammography images as either benign or malignant. The models underwent fine-tuning on the Digital Database for Screening Mammography (DDSM) dataset, utilizing pre-trained weights derived from ImageNet. Among the architectures evaluated, ResNet50 demonstrated superior performance, achieving an accuracy of 88%, precision of 85%, recall of 90%, and a ROC AUC of 0.92, outpacing both VGG16 and InceptionV3 in all comprehensive metrics. These findings highlight the effectiveness of transfer learning in enhancing breast cancer detection by improving diagnostic accuracy and reliability.

A sophisticated deep ensemble transfer learning model, combined with a neural network classifier, was proposed by Arora et al. in [28] for the automated extraction of features and classification of mammographic images. This approach involved the pre-processing of images, the extraction of robust features using the ensemble model, and the optimization of these features into a cohesive vector for classification. The neural network classifier successfully distinguished between benign and malignant tumors, achieving an accuracy of 88% and an AUC of 0.88, thereby demonstrating the promising capabilities of this robust computer-aided diagnosis (CADx) system for breast cancer classification.

Aguerchi et al. [7] developed a highly accurate convolutional neural network (CNN) model for breast cancer detection through mammographic imaging. The proposed methodology is based on the Particle Swarm Optimization (PSO) algorithm, which helps identify optimal hyperparameters and structural configurations for the CNN model. The CNN model utilizing PSO achieved impressive success rates of 98.23% and 97.98% on the DDSM and MIAS datasets, respectively.

Histopathological images: Mansour [29] introduced a computer-assisted system for breast cancer detection, employing an adaptive learning-based Gaussian Mixture Model (GMM) alongside feature extraction using AlexNet-DNN, complemented by principal component analysis (PCA) and linear discriminant analysis (LDA). The proposed approach achieved a performance score of 96.70% for the AlexNet-FC7 model using the BreakHis dataset.

An evaluative analysis was conducted on the efficacy of CNNs in conjunction with four widely recognized CNN-based architectures: VGG16, VGG19, MobileNet, and ResNet50, for the classification of breast cancer using histopathological images from the BreakHis dataset. Among the evaluated classifiers, VGG16 exhibited superior performance, achieving an accuracy of 94.67%, precision of 92.60%, F1-score of 85.21%, and recall of 80.52%. These results substantiate its effectiveness in the classification of malignant versus non-malignant tumors, as noted by Agarwal et al. [30].

Spanhol et al. [31] published a dataset containing 7,909 breast cancer histology images, classified into normal and cancerous categories. The primary objective of this dataset is to facilitate the automatic classification of these images into two groups, providing medical professionals with a useful computer-aided diagnosis tool. Their analysis achieved an accuracy of 80% to 85%, indicating room for improvement. The researchers employed machine learning algorithms such as KNN, SVM, quadratic linear analysis, and random forest for feature analysis.

Zhou et al. [4] utilized a three-dimensional DCNNs detect and localize breast cancer in dynamic contrast-enhanced MRI datasets. Despite being a relatively under-trained model, the 3D-CNN achieved an accuracy of 83.7%, showcasing the potential of CNNs for MRI-based breast cancer detection.

Yurttakal et al. [32] developed a multilayer convolutional neural network (CNN) that used pixel information and on-line data enhancement to detect malignant or benign lesions in MRI images. Their model demonstrated impressive performance, achieving an accuracy of 98.33%, which underscores the potential of pixel-based feature extraction and data augmentation to improve model efficacy.

In the field of ultrasound imaging, Ragab et al. [33] aimed to identify and classify breast cancer using an innovative ensemble deep learning-based clinical decision support system. To accurately identify tumor-affected regions, the researchers developed an optimal multilevel thresholding technique for image segmentation. In addition, they established a feature extraction ensemble comprising three distinct deep learning models, combined with an effective machine learning classifier for breast cancer diagnosis.

Eroğlu Y [34] designed a hybrid CNN system that utilizes ultrasonography images for breast cancer diagnosis. This system extracts features from AlexNet, MobileNetV2, and ResNet50, concatenating these features and applying the mRMR (minimum Redundancy Maximum Relevance) feature selection method to identify the most significant features. The classification was performed using SVM and k-NN classifiers, leading to an outstanding accuracy rate of 95.6

One study [35] utilized two breast ultrasound datasets from different platforms, with breast ultrasound images as the primary dataset. The BUSI dataset contains 780 images, including 133 normal, 210 malignant, and 437 benign specimens. Another dataset (referred to as dataset B) includes 163 images comprising 110 normal and 53 malignant specimens. To improve the dataset, generative adversarial networks (GANs) were used to augment the data. The study explored the classification of breast ultrasound images using deep learning (DL), comparing CNN-AlexNet and transfer learning approaches, both with and without data augmentation. Over 60 training epochs with a learning rate of 0.0001, their model achieved an accuracy of 94% for the BUSI dataset, 92% for dataset B, and 99% when data augmentation was applied.

## 3. Material and Methods

### 3.1. Datasets Used

Deep learning models in breast cancer research depend on diverse and high-quality datasets. In this study, we utilized publicly available breast cancer datasets from multiple imaging modalities. For mammography, we used the MIAS, DDSM, and INbreast datasets. We also incorporated public ultrasound imaging datasets. Magnetic resonance imaging (MRI) was performed using a publicly available breast imaging dataset. Lastly, for histopathology images, we employed the BreaKHis dataset. Our proposed method was thoroughly tested across these imaging modalities to evaluate its capacity to distinguish benign from malignant findings using these diverse datasets.

DDSM: The Digital Database for Screening Mammography (DDSM) [36] is a crucial resource for breast cancer research. It includes mammography images from screening programs, classified as benign, malignant, and normal. Patient metadata, including age, breast density, and pathology findings, enhances the dataset for developing and validating breast cancer identification algorithms. The richness and high resolution of this dataset make it a reliable baseline for testing diagnostic models in clinical settings.

MIAS: The Mammographic Image Analysis Society (MIAS) [36] database is a publicly available dataset of mammographic images with detailed annotations. MIAS is highly useful for automated breast cancer detection as it contains well-defined annotations and includes both normal and abnormal cases, facilitating the development of algorithms for early breast cancer detection.

INbreast: The high-resolution, full-field digital mammography database INbreast [36] contains annotated mammograms of various types. Benign or malignant abnormalities are classified according to craniocaudal (CC) and mediolateral oblique (MLO) views. The extensive annotations in this collection help to locate and characterize lesions. Due to its high image quality and substantial metadata, INbreast is widely used for machine learning research in breast cancer diagnosis.

Breast Ultrasound Images Dataset: [35] Available on Kaggle, this dataset contains 971 breast ultrasound images originally categorized into three classes: normal, benign, and malignant, with corresponding labels (0, 1, and 2). For the purposes of this study, the “normal” images were excluded, and only benign and malignant images were used for classification. The dataset thus contains two classes (benign = 0, malignant = 1), as reflected in all tables and analyses in the manuscript. This preprocessing ensures a focused evaluation of the model for distinguishing between benign and malignant findings.

MRI Datasets: Magnetic Resonance Imaging (MRI) [37] datasets provide complex 3D breast tissue models essential for breast cancer research. Contrast-enhanced MRI images are more effective than standard mammography at detecting small abnormalities in dense breast tissues. These datasets are critical for lesion characterization, tumor detection, and staging investigations, enhancing the diagnostic precision of machine learning models in complex clinical scenarios.

BreaKHis: The Breast Cancer Histopathological Database (BreaKHis) [38] contains 7909 microscopic breast tissue images from 82 individuals. The images, captured at magnifications of 40×, 100×, 200×, and 400×, are classified as benign or malignant. This dataset is essential for histopathological image processing research, supporting the development of cellular-level breast cancer subtype classification algorithms. The focus on pathology enhances the diagnostic accuracy and robustness of machine learning models.

**Figure 1:**
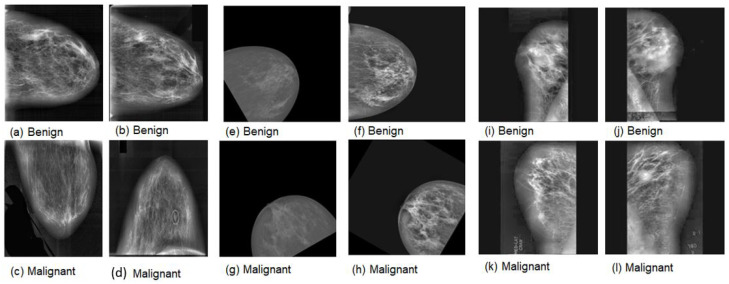
Samples of mammography images. (**a**–**d**) DDSM; (**e**–**h**) INbreast; (**i**–**l**) MIAS.

**Figure 2:**
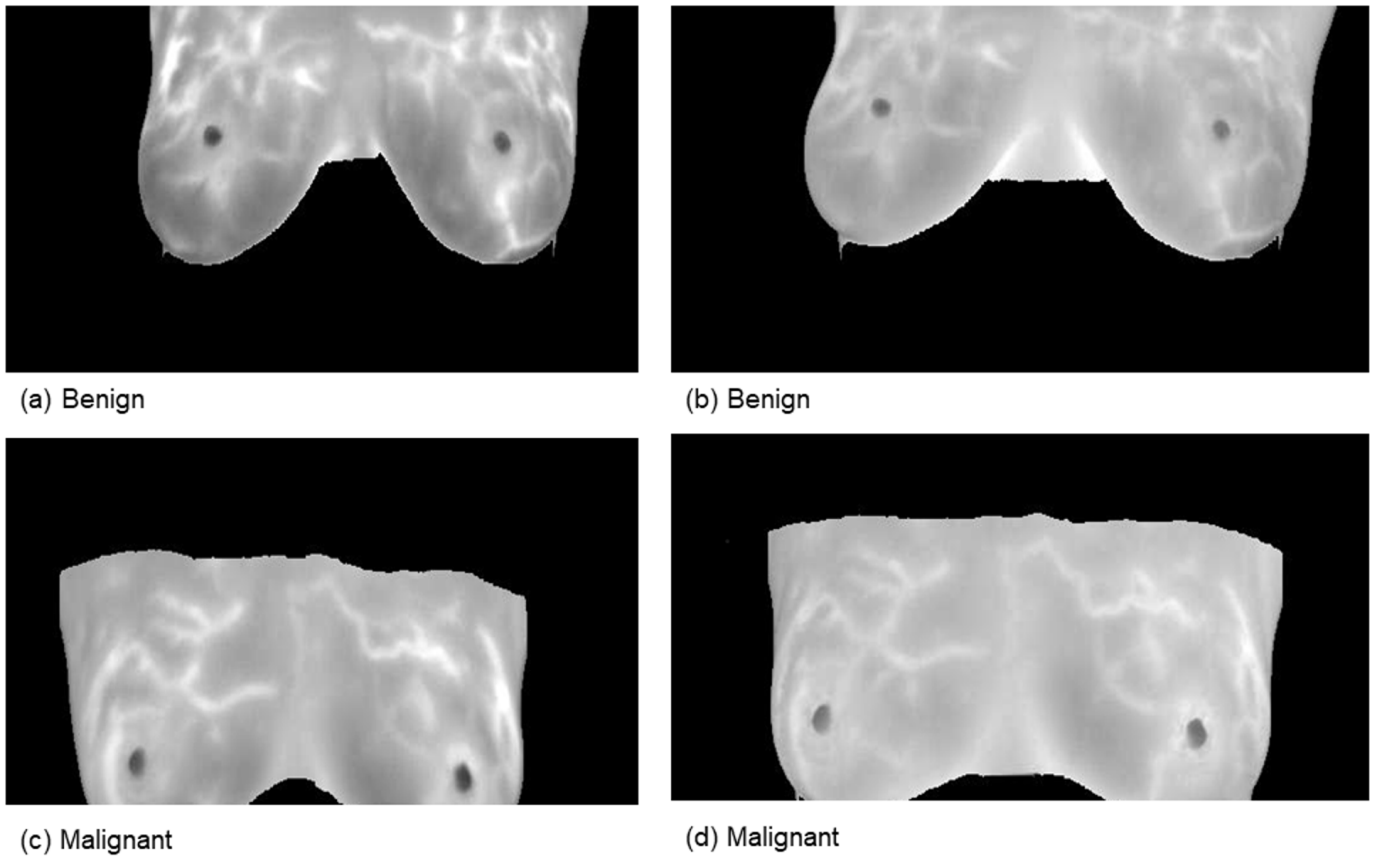
Samples from the magnetic resonance imaging (MRI) dataset.

**Figure 3:**
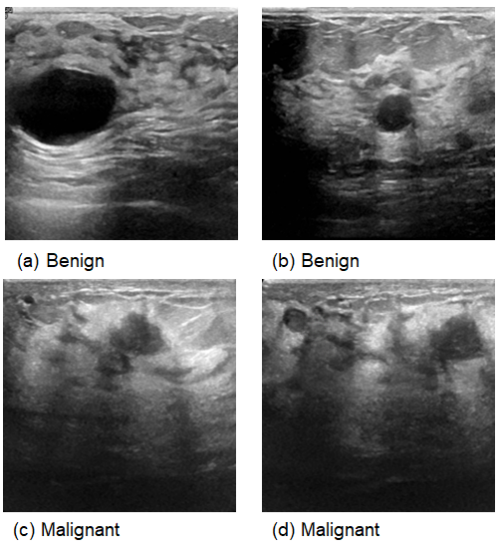
Samples from Ultrasound images.

**Figure 4:**
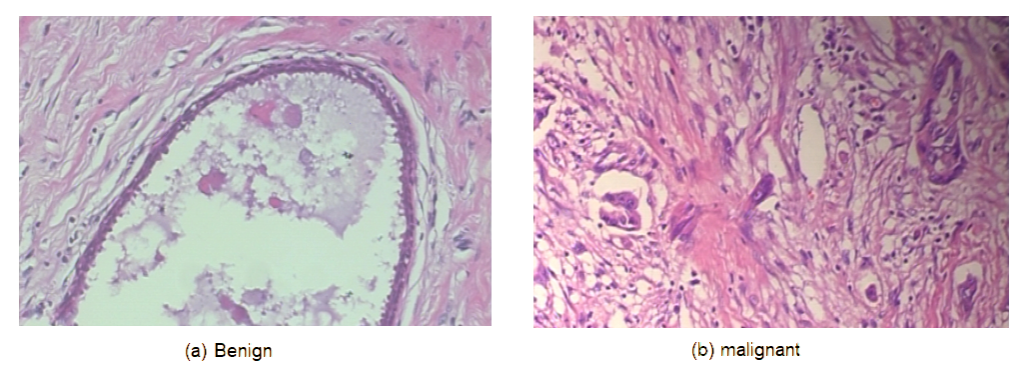
Samples from the BreakHis dataset.

This comprehensive approach of utilizing datasets from multiple imaging modalities shown in Figure 1, Figure 2, Figure 3 and Figure 4 and summarized in Table 1, including mammography, ultrasound, MRI, and histopathological images, ensures robust model validation and enhances the system’s capacity to accurately classify benign and malignant findings. By leveraging these diverse datasets, we aim to develop a more generalizable and effective breast cancer classification system.

### 3.2. Proposed Methodology

**Figure 5:**
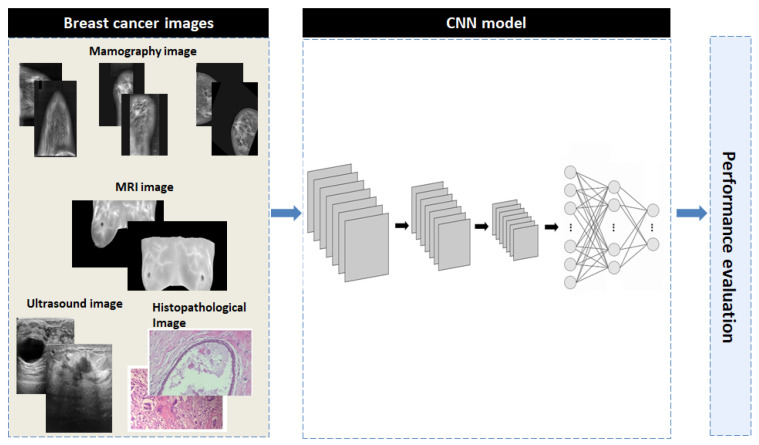
The overall block diagram of the proposed CNN model.

The proposed methodology consists of three phases: image preprocessing, training of an advanced convolutional neural network (CNN) model using multiple imaging datasets and the performance evaluation. Figure 5 provides a concise overview of the proposed methodology to aid in understanding.

- Preprocessing: All images were resized to 128 × 128 pixels and normalized by dividing pixel values by 255.
- Data Augmentation: Random rotations, horizontal flips, and zoom were applied during training.
- Dataset Splits: 5-fold cross-validation was used for all datasets with fixed random seeds ensuring reproducibility.

#### 3.2.1. Image Preprocessing

In this study, we prepare the data for deep learning models using image preprocessing techniques. Contrast Limited Adaptive Histogram Equalization (CLAHE) was applied to enhance image contrast, highlighting critical features such as masses and calcifications. To ensure consistency for the neural network, all images were resized to 128 × 128 pixels. Since mammography scans are monochromatic, the images were adjusted to facilitate the data collection process. These preprocessing techniques improve the model’s ability to detect and classify breast cancer.

#### 3.2.2. Convolutional Neural Networks

Convolutional Neural Networks (CNNs) have emerged as a highly effective deep learning architecture for medical image processing, including breast cancer prediction and diagnosis. CNNs can successfully analyze imaging modalities such as mammograms, ultrasounds, MRI scans and hispathology, which are crucial for the early detection of breast cancer.

CNNs improve predictive accuracy by integrating multiple imaging datasets, offering a powerful tool for comprehensive breast cancer investigation. This deep learning-based approach not only enhances diagnostic performance but also facilitates personalized treatment planning, significantly impacting clinical decision-making and patient outcomes.

### 3.3. Proposed CNN Model

The proposed CNN architecture consists of three convolutional layers and a single fully connected layer, chosen to balance model complexity, computational efficiency, and classification performance. Convolutional and max-pooling layers extract hierarchical features, capturing progressively more complex patterns. The fully connected layer integrates high-level features, and the final activation function performs binary classification between benign and malignant cases. This streamlined design minimizes parameters and computational load while maintaining robust feature extraction and accurate classification, addressing both efficiency and performance considerations.

#### 3.3.1. Architecture of Proposed CNN Model

**Figure 6:**
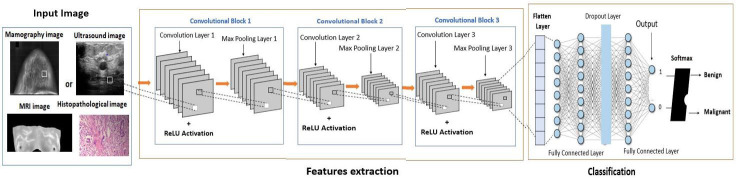
The comprehensive architecture of the proposed CNN architecture.

The proposed CNN architecture (shown in Figure 6) consists of three convolutional layers, a max-pooling layer, and a fully connected binary classification layer. The model begins with a Conv2D layer containing 32 filters, a kernel size of (3, 3), and ReLU activation, designed to process grayscale input. Subsequent convolutional layers extract more complex features with 64 and 128 filters, while max-pooling layers reduce spatial dimensions and computational costs. After flattening, a dense layer with 128 neurons and ReLU activation encodes high-level representations, followed by a dropout layer with a rate of 0.5 to prevent overfitting. Finally, a sigmoid activation function produces a single output neuron, classifying the input as either benign or malignant. This architectural framework prioritizes simplicity and efficiency, enabling rapid learning with high accuracy and minimal computational load.

#### 3.3.2. Learning Method

The proposed convolutional neural network (CNN) architecture employs the Adam optimization method for adaptive learning rate adjustments and uses a binary cross-entropy loss function for binary classification tasks, ensuring rapid convergence. The model was trained for 80 epochs with a batch size of 32, utilizing a novel data generator to enhance variability and generalization. A detailed training history was recorded for each epoch to monitor performance and detect potential overfitting. The model’s classification performance was evaluated using four key metrics: accuracy, Precision, Recall and F1 score. The metrics are defined as follows:

(1) $$Accuracy=\frac{TP+TN}{TP+FP+FN+TN}$$

(2) $$Precision=\frac{TP}{TP+FP}$$

(3) $$Recall=\frac{TP}{TP+FN}$$

(4) $$F1-score=2*\frac{Precision*Recall}{Precision+Recall}$$

**Figure 7:**
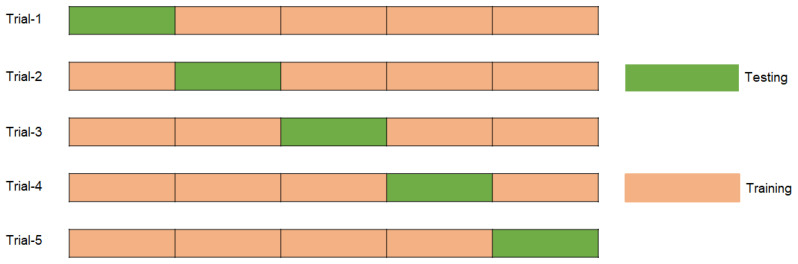
Distribution of samples for each trial by using 5-fold cross validation.

FP, FN, TN, and TP represent false positives, false negatives, true negatives, and true positives, respectively. Using these criteria, a comprehensive evaluation of the model’s classification performance in both categories enhances precision and reliability. The model was evaluated using stratified 5-fold cross-validation (CV), where the dataset was divided into five subsets while maintaining the original class distribution of benign and malignant cases. In each iteration, one subset was reserved for testing, and the remaining subsets were used for training. To further improve robustness and mitigate potential effects of class imbalance, the entire CV process was repeated three times, ensuring consistent and balanced evaluation across all folds. Figure 7 illustrates the sample distribution of the 5-fold cross-validation for each iteration.

The proposed CNN strikes a balance between performance and efficiency by utilizing three convolutional layers for feature extraction and a single dense layer to prevent overfitting and manage complexity. ReLU activation, a 0.5 dropout rate, and 0.0001 L2 regularization are incorporated to enhance learning and generalization. A batch size of 32, 80 epochs, and a carefully tuned learning rate of 0.0001 ensure stable optimization dynamics, while the Adam optimizer accelerates convergence. The combination of binary cross-entropy and a sigmoid output function guarantees accurate binary classification. Model performance is evaluated using accuracy, Precision, Recall and F1 score, while 5-fold cross-validation enhances reliability and generalizability. These methodological choices result in a robust and efficient medical imaging model.

## 4. Experiments and Results

### 4.1. Experimental Setting

The experiments were conducted on Kaggle Notebooks with dual NVIDIA Tesla T4 GPUs (16 GB each), 32 GB RAM, and 100 GB storage, providing enhanced performance for large-scale deep learning tasks. The models were implemented in Python 3.10 within this flexible and scalable environment.

### 4.2. Results and Analysis

#### 4.2.1. Results of Proposed Method for Mammography Datasets

##### Results of Proposed Method for MIAS Dataset

**Figure 8:**
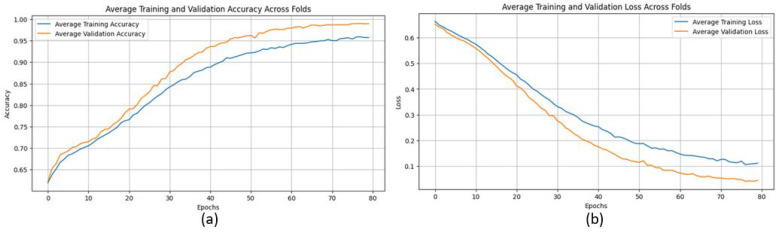
(**a**) Average Training accuracy Across Folds of the proposed CNN model versus number of epochs with MIAS dataset; (**b**) Average Training Loss Across Folds of the proposed CNN model versus number of epochs with MIAS dataset.

**Figure 9:**
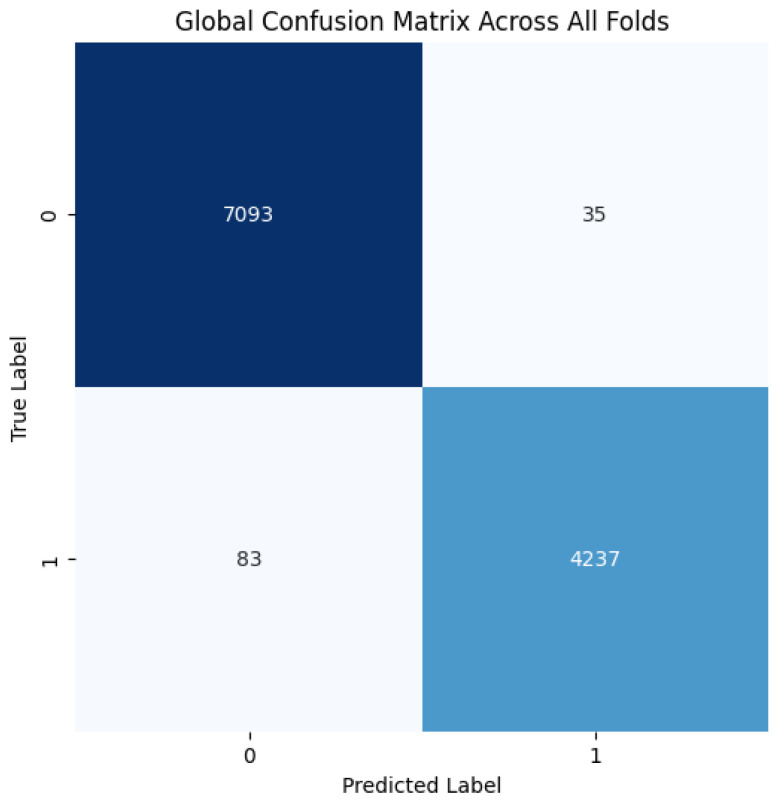
Confusion matrix across all folds for MIAS dataset.

The proposed CNN model yielded impressive results on the MIAS dataset, achieving an average accuracy of 98.97%, precision of 99.20%, recall of 98.11%, and an F1 score of 98.64% across three runs using 5-fold cross-validation. The model effectively classifies mammographic images, with consistent precision and recall metrics, underscoring its reliability in identifying both positive and negative instances. The low variability across folds indicates the model’s stability and generalization. Small fluctuations in metrics, such as a slight decrease in recall in some folds, suggest potential areas for optimization to enhance sensitivity. The detailed performance metrics are summarized in Table 2. Training curves showing the average accuracy and loss across folds are illustrated in Figure 8, while the confusion matrix aggregated across all folds is presented in Figure 9. Overall, these results highlight the model’s strong potential for breast cancer classification and diagnostics.

##### Results of the Proposed Method for DDSM Dataset

**Figure 10:**
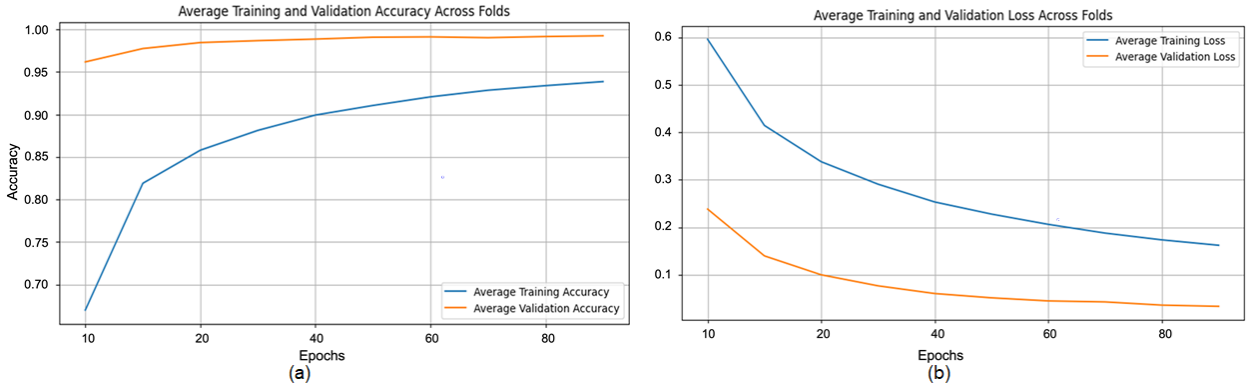
(**a**) Average Training accuracy Across Folds of the proposed CNN model versus number of epochs with DDSM dataset; (**b**) Average Training Loss Across Folds of the proposed CNN model versus number of epochs with DDSM dataset.

**Figure 11:**
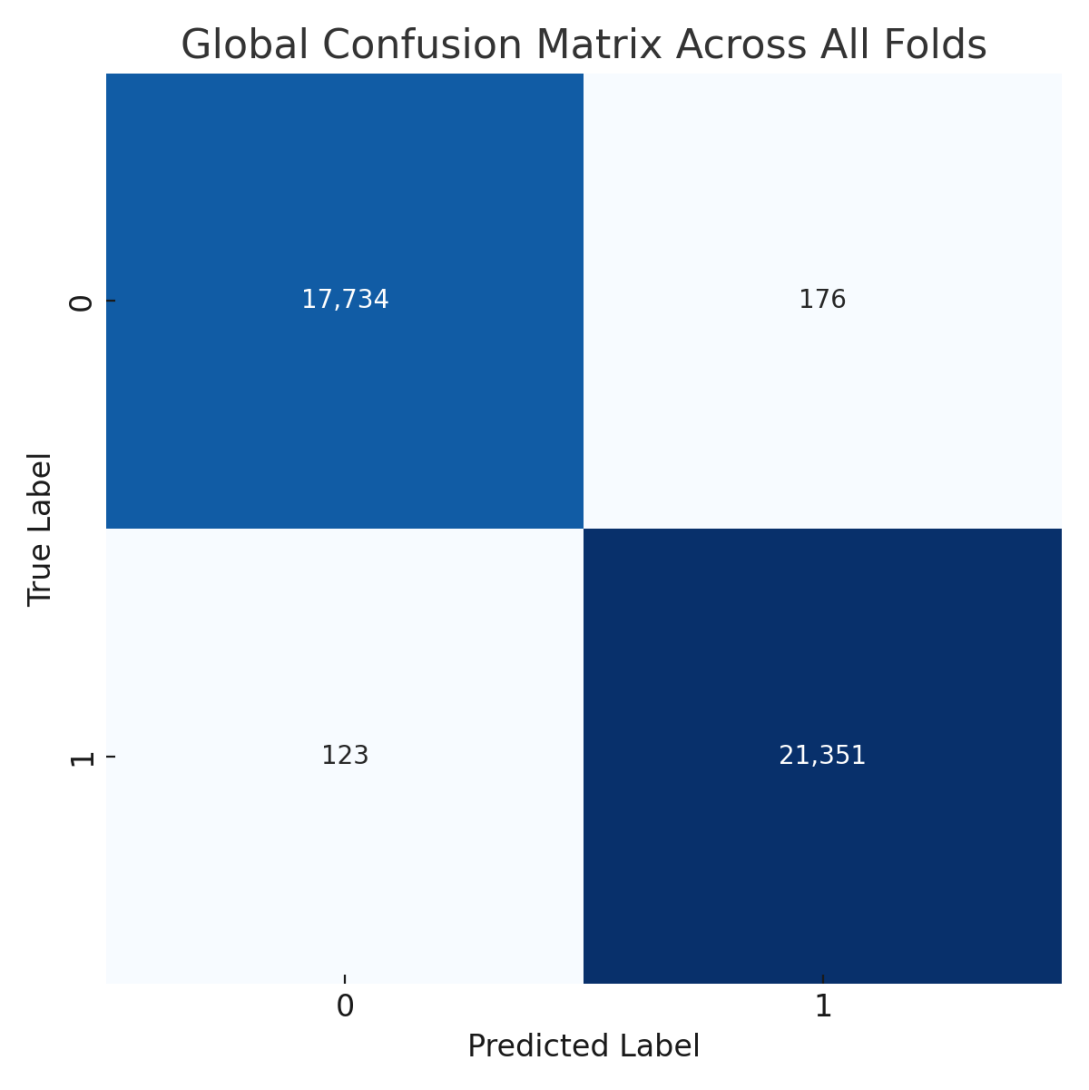
Confusion matrix across all folds for DDSM dataset.

The proposed CNN model demonstrates remarkable robustness and performance on the DDSM dataset, achieving an overall average accuracy of 99.24%, precision of 99.20%, recall of 99.43%, and an F1 score of 99.31% across three runs using 5-fold cross-validation. These results highlight the model’s ability to effectively classify mammographic images, with consistent precision and recall metrics, reflecting its reliability in identifying both benign and malignant instances. The minimal variation across folds underscores the model’s stability and generalization capabilities. Slight discrepancies in memory usage across individual folds suggest potential areas for optimization, particularly to enhance sensitivity. The detailed performance metrics are summarized in Table 3. Training curves showing the average accuracy and loss across folds are illustrated in Figure 10, while the confusion matrix aggregated across all folds is presented in Figure 11. Overall, these findings affirm the model’s strong potential for breast cancer classification and emphasize its diagnostic value.

##### Results of Proposed Method for INbreast Dataset

**Figure 12:**
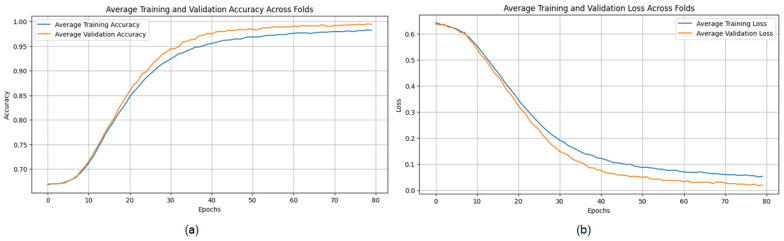
(**a**) Average Training accuracy Across Folds of the proposed CNN model versus number of epochs with INbreast dataset; (**b**) Average Training Loss Across Folds of the proposed CNN model versus number of epochs with INbreast dataset.

**Figure 13:**
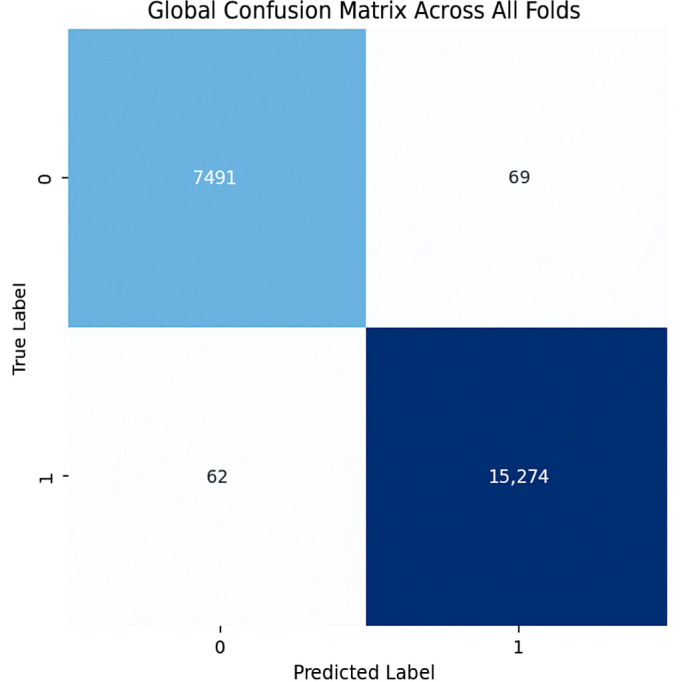
Confusion matrix across all folds for INbreast dataset.

The evaluation of the proposed CNN model on the INbreast dataset highlights its effectiveness and outstanding performance, achieving an average accuracy of 99.43%, precision of 99.55%, recall of 99.60%, and an F1 score of 99.57% across three runs using 5-fold cross-validation. These results emphasize the model’s strong ability to accurately classify breast cancer images, with consistent precision and recall metrics confirming its reliability in identifying both malignant and benign instances. The minimal performance variation across the different folds further highlights the model’s stability and generalization capability. Small fluctuations in metrics, such as a slight decrease in recall in certain folds, suggest potential areas for improvement to boost sensitivity. The detailed performance metrics are summarized in Table 4. Training curves showing the average accuracy and loss across folds are illustrated in Figure 12, while the confusion matrix aggregated across all folds is presented in Figure 13. Overall, these results validate the model’s efficacy in breast cancer classification and enhance its potential as a reliable diagnostic tool.

#### 4.2.2. Results of Proposed Method for Ultrasound Datasets

The proposed CNN model performs well on the Ultrasound dataset, achieving an average accuracy of 98.00%, precision of 98.11%, recall of 97.84%, and an F1 score of 97.97% across three iterations using 5-fold cross-validations (Table 5). These metrics demonstrate the model’s ability to classify ultrasound images with consistent precision and recall values, effectively distinguishing between benign and malignant cases. The limited variation across folds highlights the model’s stability and generalization. However, slight differences in memory usage, particularly in certain folds, suggest the need for further tuning to enhance sensitivity. Overall, the results reinforce the model’s potential for breast cancer classification and its diagnostic capabilities.

**Figure 14:**
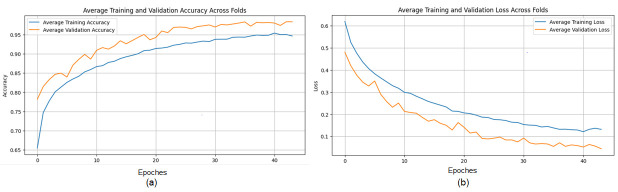
(**a**) Average Training accuracy Across Folds of the proposed CNN model versus number of epochs with Ultrasound dataset; (**b**) Average Training Loss Across Folds of the proposed CNN model versus number of epochs with Ultrasound dataset.

**Figure 15:**
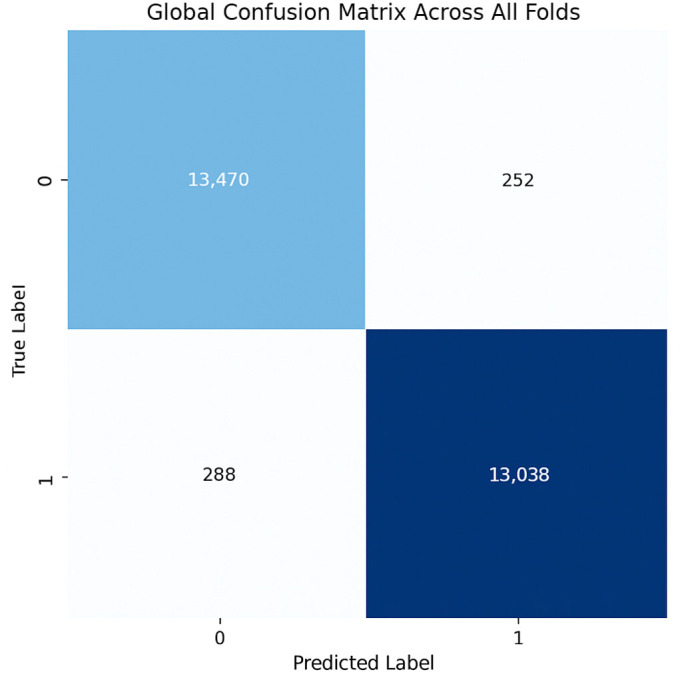
Confusion matrix across all folds for Ultrasound dataset.

The training process shows consistent convergence across folds, as illustrated by the average training accuracy and loss curves (Figure 14). The confusion matrix confirms the model’s effectiveness in correctly classifying most cases, with only a small number of misclassifications observed (Figure 15).

#### 4.2.3. Results of the Proposed Method on Magnetic Resonance Imaging (MRI) Datasets

The evaluation of the proposed Convolutional Neural Network (CNN) architecture on the Magnetic Resonance Imaging (MRI) dataset highlights its robustness and outstanding performance, achieving an average accuracy of 98.43%, precision of 98.27%, recall of 98.63%, and an F1 score of 98.45% across three iterations using a five-fold cross-validation methodology. These metrics demonstrate the model’s ability to accurately classify MRI images, with consistent precision and recall rates indicating its potential to effectively differentiate between benign and malignant conditions. The model’s stability and applicability are evidenced by the minimal variability across folds. However, slight differences in memory usage across specific folds suggest opportunities to further enhance sensitivity. Overall, the results affirm the model’s capability for breast cancer classification and its potential as a reliable diagnostic tool.

The evaluation of the proposed Convolutional Neural Network (CNN) architecture on the Magnetic Resonance Imaging (MRI) dataset highlights its robustness and outstanding performance, achieving an average accuracy of 98.43%, precision of 98.27%, recall of 98.63%, and an F1 score of 98.45% across three iterations using a five-fold cross-validation methodology (Table 6). These metrics demonstrate the model’s ability to accurately classify MRI images, with consistent precision and recall rates indicating its potential to effectively differentiate between benign and malignant conditions. The model’s stability and applicability are evidenced by the minimal variability across folds. However, slight differences in memory usage across specific folds suggest opportunities to further enhance sensitivity. Overall, the results affirm the model’s capability for breast cancer classification and its potential as a reliable diagnostic tool.

**Figure 16:**
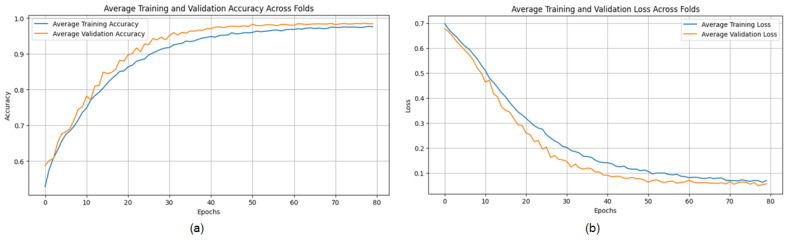
(**a**) Average Training accuracy Across Folds of the proposed CNN model versus number of epochs with MRI dataset; (**b**) Average Training Loss Across Folds of the proposed CNN model versus number of epochs with MRI dataset.

**Figure 17:**
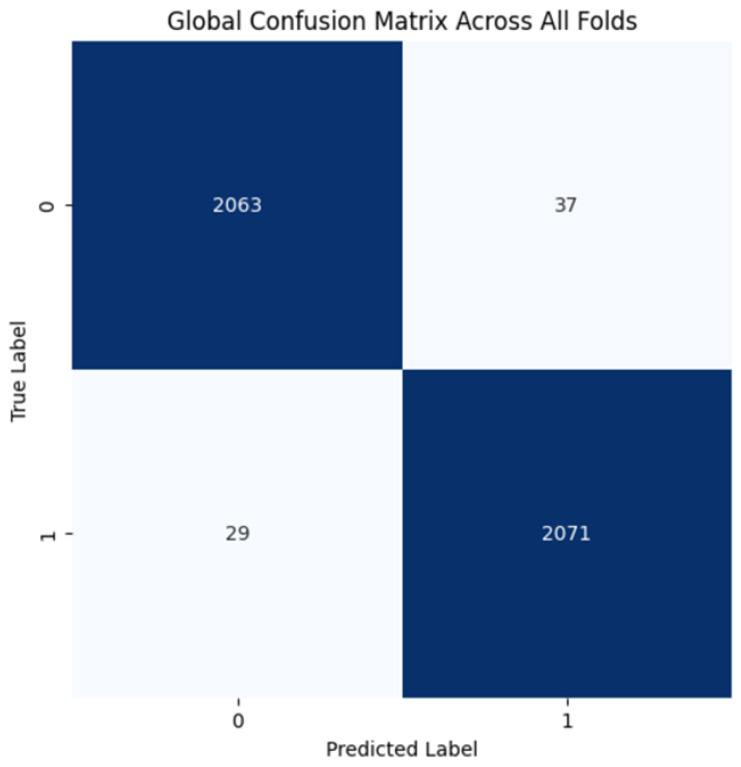
Confusion matrix across all folds for MRI dataset.

The training process shows consistent convergence across folds, as indicated by the average training accuracy and loss curves (Figure 16). The confusion matrix further demonstrates the model’s ability to correctly classify most cases, with minimal misclassifications observed across folds (Figure 17).

#### 4.2.4. Results of the Proposed Method on Histopathological Datasets

The model demonstrates robust performance across all folds, achieving an average accuracy of 0.863, precision of 0.879, recall of 0.929, and an F1 score of 0.903, indicating an effective balance between identifying positive cases and minimizing false positives (Table 7). The high recall highlights the model’s proficiency in detecting positives, which is crucial in fields like medical diagnostics. While precision is generally high, slight variations in accuracy across folds and minor changes in precision (e.g., 0.868 in Fold 1, Run 1) suggest opportunities to further reduce false positives and improve consistency. Overall, the model shows strong performance and robustness, with potential for fine-tuning to enhance precision and uniformity.

**Figure 18:**
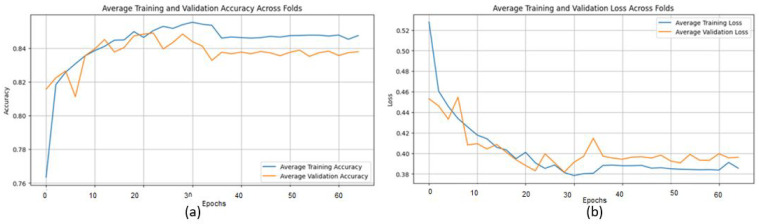
(**a**) Average Training accuracy Across Folds of the proposed CNN model versus number of epochs with BreaKHis dataset; (**b**) Average Training Loss Across Folds of the proposed CNN model versus number of epochs with BreaKHis dataset.

**Figure 19:**
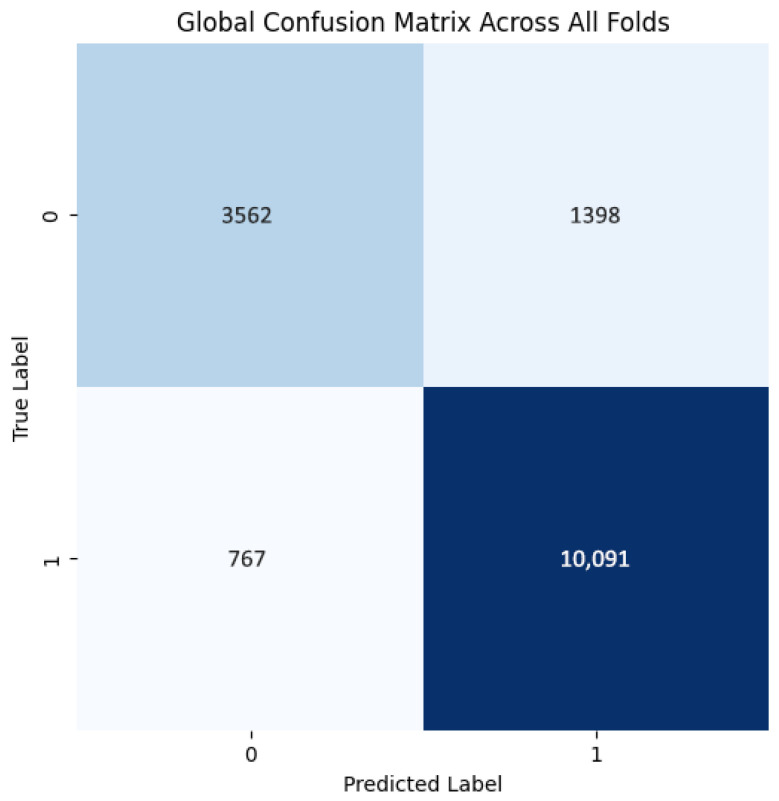
Confusion matrix across all folds for Histopathological dataset.

The training curves show stable convergence across folds, as illustrated by the average training accuracy and loss (Figure 18). The confusion matrix further confirms the model’s ability to classify cases correctly, with only a few misclassifications observed across folds (Figure 19). These figures complement the metrics reported in Table 7.

### 4.3. Comparative Analysis with Existing CAD Models

#### 4.3.1. Comparative Analysis with Existing CAD Models on Mammography Datasets

Table 8 shows that the CNN model outperforms traditional methods in classification accuracy on the DDSM, MIAS, and INbreast datasets. The model achieves 99.2% accuracy on DDSM, 98.97% on MIAS, and 99.43% on INbreast. In comparison, Chougrad et al.’s ensemble, which includes VGG16, ResNet50, and Inception v3, achieved accuracies of 97.35% on DDSM and 98.23% on MIAS [21]. Similarly, Muduli et al.’s DeepCNN reached 90.68% on DDSM, 96.55% on MIAS, and 91.28% on INbreast [25], while Rouhi and Jafari’s MLP recorded 88.61% on DDSM, 90.94% on MIAS, and 89.23% on INbreast [26]. Jafari et al.’s feature-extraction technique achieved 96% accuracy on DDSM and 94.5% on MIAS [18], and Rahman et al. reported accuracies of 85.7% for ResNet50 and 79.6% for Inception v3 on DDSM [22]. Lastly, Dong et al.’s enhanced kNN achieved scores of 86.54% on DDSM, 95.76% on MIAS, and 89.30% on INbreast [27]. These comparisons highlight the robustness and generalizability of the proposed CNN model, demonstrating it as a significant improvement in computer-aided diagnosis (CAD) systems for breast cancer and a valuable tool for enhancing early detection accuracy in clinical environments.

#### 4.3.2. Comparative Analysis with Existing CAD Models on Ultrasound Datasets

The results presented in Table 9 show that the proposed CNN model achieves an accuracy of 98.00% on the Breast Ultrasound Dataset, outperforming Ragab et al.’s ensemble of SqueezeNet, VGG-16, and VGG-19 models optimized through Cat Swarm Optimization and Multilayer Perceptron, which achieved 97.09% [32], as well as Eroğlu Y’s hybrid-based CNN system, which reached 95.6% [34]. These findings highlight the superior performance of the proposed CNN, demonstrating its ability to effectively balance simplicity and accuracy, surpassing more complex ensemble and hybrid approaches. The model’s proficiency in extracting meaningful features from ultrasound images is reflected in its higher accuracy, making it a promising and effective solution for breast cancer screening. However, as the evaluation was conducted on a single dataset, further validation across a wider range of datasets is recommended to assess its generalizability and robustness.

#### 4.3.3. Comparative Analysis with Existing CAD Models on Magnetic Resonance Imaging (MRI) Datasets

The proposed CNN model achieves an accuracy of 98.43% on MRI datasets, outperforming Zhou et al.’s 3D deep CNN, which achieved 83.7%, and slightly surpassing Yurttakal et al.’s multilayer CNN, which reached 98.33%, as shown in the results presented in the Table 10. The modest improvement over Yurttakal et al.’s method highlights the effectiveness of the proposed approach in optimizing feature extraction and maximizing classification performance for MRI data [32]. Furthermore, the significant improvement over Zhou et al.’s 3D deep CNN emphasizes the model’s ability to capture essential imaging features without the added complexity of 3D structures [4]. These results underscore the potential of the proposed CNN as a reliable tool for MRI data interpretation in clinical settings. However, additional validation on different MRI datasets is necessary to confirm the model’s generalizability and robustness.

#### 4.3.4. Comparative Analysis with Existing CAD Models on Histopathological Datasets

The BreakHis dataset reveals notable performance disparities in classification as shown in Table 11. Mansour [29] achieved AlexNet’s best accuracy of 96.70%, demonstrating its effectiveness for breast cancer categorization. Using CNN-based models (VGG16, VGG19, MobileNet, and ResNet 50), Agarwal et al. [30] reached an accuracy of 94.67%, which is effective but did not surpass AlexNet. Spanhol et al. [31] achieved 85% accuracy using classical classifiers (KNN, SVM, RF), highlighting that deep learning methods outperform traditional image classification techniques. Similar to Spanhol et al. [31], our CNN-based model achieved 86.42% accuracy, which, while effective, lags behind the top-performing deep learning models. We may consider modifying our CNN architecture or exploring hybrid approaches to reduce the performance gap and further enhance accuracy.

### 4.4. General Discussion

The proposed CNN model demonstrates strong performance across multiple imaging modalities, including mammography, ultrasound, MRI, and histopathology datasets, achieving accuracy rates of 99.2% on DDSM, 98.97% on MIAS, 99.43% on INbreast, 98.00% on ultrasound, and 98.43% on MRI. In contrast, performance on the BreaKHis histopathology dataset was lower, with an accuracy of 86.42%, indicating potential limitations when dealing with higher intra-class variability or complex tissue structures. While these results surpass several state-of-the-art approaches, such as those reported by Chougrad et al. [21], Muduli et al. [25], and Mansour [29], they also highlight the challenges of generalizing across heterogeneous datasets.

**Figure 20:**
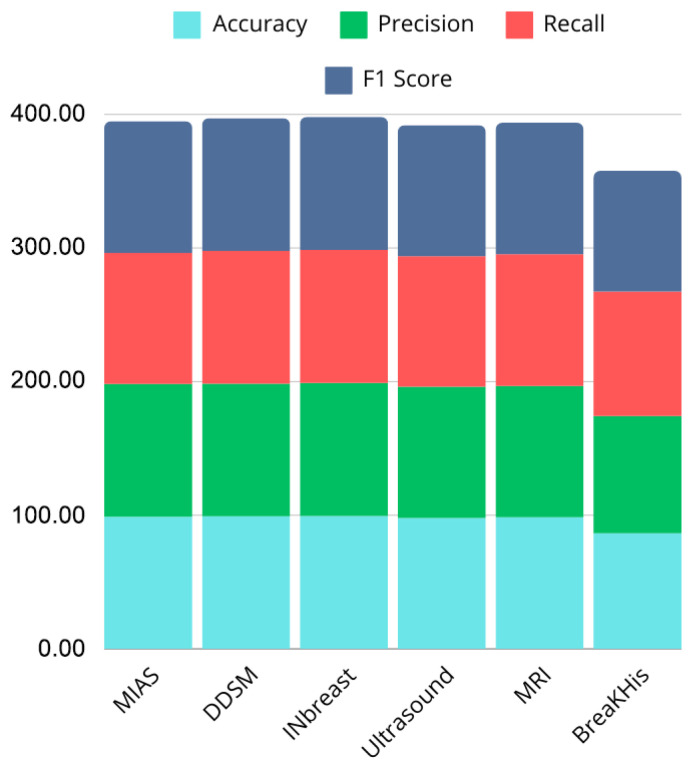
Bar chart showing performance comparison across datasets.

**Figure 21:**
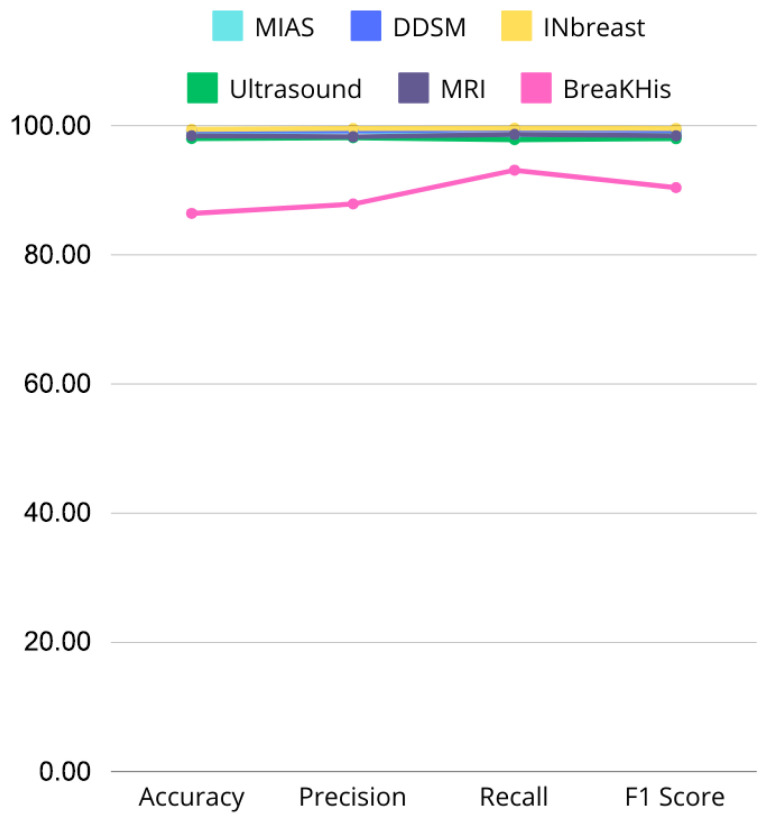
Line graph comparing Accuracy, Precision, Recall, and F1 Score across MIAS, DDSM, INbreast, Ultrasound, and MRI datasets, highlighting performance variations.

Figure 20 and Figure 21 illustrate performance comparisons across datasets and critical metrics (Accuracy, Precision, Recall, F1 Score), revealing consistent performance on most modalities but noticeable drops on histopathological images. This suggests that while the model effectively captures discriminative features in mammography, ultrasound, and MRI, additional architectural adaptations or domain-specific pre-processing may be required for histopathology tasks.

The simplicity of the CNN architecture, avoiding complex ensembles or hybrid models, contributes to computational efficiency and scalability. However, this design choice may also limit the model’s ability to capture more subtle or high-dimensional features in certain datasets, such as BreaKHis. Nonetheless, the model reliably extracts essential imaging features and provides robust classification outcomes, demonstrating its potential as a practical tool for clinical decision support.

These findings underline the model’s versatility and potential to enhance early breast cancer detection. However, additional validation on larger, multi-center datasets is essential to confirm its generalizability and address modality-specific limitations. Future work could explore hybrid architectures, multi-scale feature extraction, or integration with clinical metadata to further improve performance, particularly in challenging histopathology datasets. Overall, the proposed CNN provides a solid foundation for advancing deep learning applications in medical imaging, bridging methodological innovation with clinical relevance.

## 5. Limitations and Future Work

This study has several limitations that should be addressed in future research. A key limitation is lower performance on the BreaKHis dataset, indicating the need for further optimization to improve its effectiveness on histopathology data, the small size of the datasets used, including MIAS, INbreast, DDSM, and Ultrasound, which, while widely used in the field, may not fully capture clinical heterogeneity. The class imbalance and limited data availability restrict the model’s ability to represent the full spectrum of breast cancer imaging, thereby limiting its generalizability. Additionally, the model’s ability to learn generalized features is impacted by image quality issues, such as noise and low resolution. To overcome these challenges, future studies should incorporate larger, more diverse datasets from multiple medical institutions, imaging modalities, and patient demographics to enhance the model’s robustness and real-world applicability. Nonetheless, there remains possibility of enhancement, especially in the histopathological field. Future research may investigate architectural improvements and hybrid models to improve performance and assure the model’s robustness across various datasets and imaging modalities. There are also opportunities for future improvements to boost model performance. One potential approach is the use of pre-trained models and transfer learning, which could enable fine-tuning on larger datasets, such as those used for general object detection or medical imaging, to improve accuracy, especially in low-data scenarios. A multi-modal model combining clinical and genomic data could further enhance prognostic capabilities by integrating patient history, biopsy results, and genetic information [39]. Finally, testing the model in real-time clinical settings and optimizing it for rapid inference would help doctors make quick, informed decisions during the diagnostic process.

## 6. Conclusions

This research presents a CNN-based approach for the automated prediction and diagnosis of breast cancer using various imaging modalities, including mammography, ultrasound, MRI, and histopathological Datasets. The model achieved exceptional accuracy rates, demonstrating its ability to identify significant features and provide reliable predictions. Its simplicity, efficiency, and adaptability make it suitable for clinical applications, especially in contrast to more complex ensemble or hybrid models. The model’s capacity to generalize across diverse imaging datasets positions it as a transformative tool in computer-aided diagnosis, offering valuable support to radiologists in the early detection of breast cancer.

While the model performs well, the study identified key limitations, particularly related to dataset size, diversity, and image quality. These factors may hinder the model’s clinical utility despite its strong performance. These findings suggest the need for further research to evaluate the model on larger, more diverse datasets, and to explore enhancements such as integrating clinical and genomic data for more personalized predictions. In conclusion, this study lays a strong foundation for deep learning-based breast cancer diagnosis. By addressing its limitations and leveraging the model’s scalability, future research can improve early detection and patient outcomes, facilitating its integration into clinical workflows.

## Figures and Tables

**Table 1:** Specification of breast cancer datasets (benign versus malignant).

Dataset	B	M
MIAS (Mammogram)	2376	1440
DDSM (Mammogram)	5970	7158
INbreast (Mammogram)	2520	5112
Ultrasound	891	421
MRI	740	740
BreaKHis (Histopathology)	2480	5429

**Table 2:** Accuracy, Precision, Recall, and F1 Score (%) of the proposed CNN model for the MIAS dataset, using a 5-fold with 3 iterations.

	Accuracy	Precision	Recall	F1 Score
Run 1, Fold 1	0.989529	0.992453	0.977695	0.985019
Run 1, Fold 2	0.990826	0.989547	0.986111	0.987826
Run 1, Fold 3	0.996068	0.993080	0.996528	0.994801
Run 1, Fold 4	0.992136	0.982639	0.996479	0.989510
Run 1, Fold 5	0.985583	0.977707	0.987138	0.982400
Run 2, Fold 1	0.994764	0.988930	0.996283	0.992593
Run 2, Fold 2	0.998689	1.000000	0.996528	0.998261
Run 2, Fold 3	0.994758	0.996503	0.989583	0.993031
Run 2, Fold 4	0.998689	0.996491	1.000000	0.998243
Run 2, Fold 5	0.984273	0.990164	0.971061	0.980519
Run 3, Fold 1	0.988220	1.000000	0.966543	0.982987
Run 3, Fold 2	0.992136	0.986207	0.993056	0.989619
Run 3, Fold 3	0.977720	0.989170	0.951389	0.969912
Run 3, Fold 4	0.992136	0.996429	0.982394	0.989362
Run 3, Fold 5	0.969856	1.000000	0.926045	0.961603
Final Avg	0.989692	0.991955	0.981122	0.986379

**Table 3:** Accuracy, Precision, Recall, and F1 Score (%) of the proposed CNN model for the DDSM dataset, using a 5-fold with 3 iterations.

	Accuracy	Precision	Recall	F1 Score
Run 1, Fold 1	0.990480	0.990264	0.992334	0.991298
Run 1, Fold 2	0.992765	0.990264	0.996501	0.993373
Run 1, Fold 3	0.996192	0.994282	0.998564	0.996418
Run 1, Fold 4	0.993524	0.996506	0.991655	0.994075
Run 1, Fold 5	0.990857	0.991126	0.992481	0.991803
Run 2, Fold 1	0.988195	0.985477	0.993031	0.989240
Run 2, Fold 2	0.996192	0.993737	0.999300	0.996511
Run 2, Fold 3	0.995430	0.996405	0.994975	0.995690
Run 2, Fold 4	0.989714	0.997883	0.983310	0.990543
Run 2, Fold 5	0.995810	0.994550	0.997949	0.996247
Run 3, Fold 1	0.995811	0.993759	0.998606	0.996177
Run 3, Fold 2	0.996192	0.997197	0.995801	0.996499
Run 3, Fold 3	0.995811	0.997122	0.994975	0.996047
Run 3, Fold 4	0.991619	0.997191	0.987483	0.992313
Run 3, Fold 5	0.977524	0.963672	0.997266	0.980181
Final Avg	0.992408	0.991962	0.994282	0.993094

**Table 4:** Accuracy, Precision, Recall, and F1 Score (%) of the proposed CNN model for the INbreast dataset, using a 5-fold with 3 iterations.

	Accuracy	Precision	Recall	F1 Score
Run 1, Fold 1	0.995416	0.994955	0.997976	0.996463
Run 1, Fold 2	0.992796	0.997087	0.992271	0.994673
Run 1, Fold 3	0.993447	0.993243	0.997093	0.995164
Run 1, Fold 4	0.993447	0.992056	0.998002	0.995020
Run 1, Fold 5	0.996723	0.997162	0.998106	0.997634
Run 2, Fold 1	0.994106	0.996954	0.993927	0.995438
Run 2, Fold 2	0.992141	0.997085	0.991304	0.994186
Run 2, Fold 3	0.997379	0.997099	0.999031	0.998064
Run 2, Fold 4	0.994758	0.998995	0.993007	0.995992
Run 2, Fold 5	0.994102	0.995270	0.996212	0.995741
Run 3, Fold 1	0.994106	0.993946	0.996964	0.995452
Run 3, Fold 2	0.994761	0.996135	0.996135	0.996135
Run 3, Fold 3	0.991481	0.989433	0.998062	0.993729
Run 3, Fold 4	0.994102	0.995010	0.996004	0.995507
Run 3, Fold 5	0.995413	0.998101	0.995265	0.996681
Final Avg	0.994279	0.995502	0.995957	0.995725

**Table 5:** Accuracy, Precision, Recall, and F1 Score (%) of the proposed CNN model for the Ultrasound dataset, using a 5-fold with 3 iterations.

	Accuracy	Precision	Recall	F1 Score
Run 1, Fold 1	0.987805	0.993174	0.982002	0.987557
Run 1, Fold 2	0.977815	0.976404	0.978604	0.977503
Run 1, Fold 3	0.987243	0.980022	0.994369	0.987144
Run 1, Fold 4	0.986689	0.987585	0.985360	0.986471
Run 1, Fold 5	0.986134	0.992027	0.979753	0.985852
Run 2, Fold 1	0.978381	0.990762	0.965129	0.977778
Run 2, Fold 2	0.981143	0.976562	0.985360	0.980942
Run 2, Fold 3	0.985580	0.986456	0.984234	0.985344
Run 2, Fold 4	0.985025	0.987542	0.981982	0.984754
Run 2, Fold 5	0.986134	0.974725	0.997750	0.986103
Run 3, Fold 1	0.930155	0.929134	0.929134	0.929134
Run 3, Fold 2	0.985580	0.990888	0.979730	0.985277
Run 3, Fold 3	0.986689	0.987585	0.985360	0.986471
Run 3, Fold 4	0.973378	0.981651	0.963964	0.972727
Run 3, Fold 5	0.982806	0.982022	0.983127	0.982574
Final Avg	0.980037	0.981103	0.978391	0.979709

**Table 6:** Accuracy, Precision, Recall, and F1 Score (%) of the proposed CNN model for the MRI dataset, using a 5-fold with 3 iterations.

	Accuracy	Precision	Recall	F1 Score
Run 1, Fold 1	0.985714	0.985714	0.985714	0.985714
Run 1, Fold 2	0.967857	0.972603	0.965986	0.969283
Run 1, Fold 3	0.985714	0.992063	0.976562	0.984252
Run 1, Fold 4	0.985714	0.992647	0.978261	0.985401
Run 1, Fold 5	0.971429	0.960265	0.986395	0.973154
Run 2, Fold 1	0.996429	1.000000	0.992857	0.996416
Run 2, Fold 2	0.978571	0.986207	0.972789	0.979452
Run 2, Fold 3	0.989286	0.984496	0.992188	0.988327
Run 2, Fold 4	0.992857	0.992754	0.992754	0.992754
Run 2, Fold 5	0.971429	0.966443	0.979592	0.972973
Run 3, Fold 1	1.000000	1.000000	1.000000	1.000000
Run 3, Fold 2	0.996429	0.993243	1.000000	0.996610
Run 3, Fold 3	0.982143	0.969466	0.992188	0.980695
Run 3, Fold 4	0.985714	0.978571	0.992754	0.985612
Run 3, Fold 5	0.975000	0.966667	0.986395	0.976431
Final Avg	0.984286	0.982743	0.986296	0.984472

**Table 7:** Accuracy, Precision, Recall, and F1 Score (%) of the proposed CNN model for the BreaKHis dataset, using a 5-fold with 3 iterations.

	Accuracy	Precision	Recall	F1 Score
Run 1, Fold 1	0.850859	0.858432	0.931476	0.893463
Run 1, Fold 2	0.857360	0.870990	0.932067	0.900494
Run 1, Fold 3	0.869499	0.873288	0.945846	0.908120
Run 1, Fold 4	0.872028	0.874751	0.953757	0.912548
Run 2, Fold 1	0.853387	0.891994	0.889307	0.890649
Run 2, Fold 2	0.862418	0.878537	0.929876	0.903478
Run 2, Fold 3	0.866464	0.866216	0.951039	0.906648
Run 2, Fold 4	0.881133	0.906582	0.925578	0.915981
Run 3, Fold 1	0.861982	0.866065	0.939759	0.901408
Run 3, Fold 2	0.864441	0.887949	0.920380	0.903874
Run 3, Fold 3	0.873040	0.898909	0.916914	0.907822
Run 3, Fold 4	0.857865	0.870383	0.936416	0.902193
Final Avg	0.864206	0.878675	0.931035	0.903890

**Table 8:** Comparative performance analysis of existing CAD models utilizing mammography datasets.

Method	Classifier	Acc (%)		
		DDSM	MIAS	INbreast
Chougrad et al. [21]	VGG16, ResNet50, Inception v3	97.35%	98.23%	-
Muduli et al. [25]	DeepCNN	90.68%	96.55%	91.28%
Rouhi et Jafari. [26]	MLP	88.61%	90.94%	89.23%
Jafari et al. [24]	Feature-Extraction-based Classification	96%	94.5%	-
Rahman et al. [22]	ResNet 50 Inception V3	85.7% 79.6%	-	-
Dong et al. [27]	Improved kNN	86.54%	95.76%	89.30%
Aguerchi et al. [7]	PSOCNN	98.23%	97.98%	-
Our proposed method	CNN	99.24%	98.97%	99.43%

**Table 9:** Comparative performance analysis of existing CAD models using Ultrasound Datasets.

Method	Classifier	Dataset	ACC
Ragab et al. [33]	SqueezeNet, VGG-16, VGG-19	Breast Ultrasound Dataset	97.09%
Eroğlu et al. [34]	Hybrid-based CNN system	Breast Ultrasound Dataset	95.6%
Our proposed method	CNN	Breast Ultrasound Dataset	98.00%

**Table 10:** Comparative performance analysis of existing CAD models using MRI Datasets.

Method	Classifier	Dataset	ACC
Zhou et al. [4]	3D deep CNN	MRI	83.7%
Yurttakal et al. [32]	Multi-layer CNN	MRI	98.33%
Our proposed method	CNN	MRI	98.43%

**Table 11:** Comparative performance analysis of existing CAD models using Histopathological Datasets.

Method	Classifier	Dataset	ACC
Mansour. [29]	AlexNet	BreakHis	96.70%
Agarwal et al. [30]	CNN-based architectures-VGG16, VGG19, MobileNet, and ResNet 50	BreakHis	94.67%
Spanhol et al. [31]	Multi-classifiers such as KNN, SVM, quadratic linear analysis, and RF	BreakHis	85%
Our proposed method	CNN	BreakHis	86.42%

## Data Availability

The original data presented in the study are openly available in the following public repositories: sciencedirect: https://doi.org/10.1016/j.dib.2020.105928 (accessed on 12 November 2024), kaggle: https://www.kaggle.com/datasets/uzairkhan45/breast-cancer-patients-mris (accessed on 12 November 2024), https://www.kaggle.com/datasets/rehelzannat/breakhis-total (accessed on 12 November 2024).

## Abbreviations

The following abbreviations are used in this manuscript:

CNN	Convolutional Neural Network
DCNNs	Deep convolutional neural networks
DL	Deep Learning
MIAS	Mammogram Image Analysis Society
DDSM	Digital Dataset for Screening Mammography
MRI	Magnetic Resonance Imaging
BreaKHis	Breast Cancer Histopathological Database
